# The Impact of Work Characteristics on Social Distancing: Implications at the Time of COVID-19

**DOI:** 10.3390/ijerph18105074

**Published:** 2021-05-11

**Authors:** Keisuke Kokubun, Yoshinori Yamakawa

**Affiliations:** 1 Economic Research Institute, Japan Society for the Promotion of Machine Industry, Tokyo 105-0011, Japan; 2 Smart-Aging Research Center, Tohoku University, Sendai 980-8575, Japan; 3 Open Innovation Institute, Kyoto University, Kyoto 606-8501, Japan; yamakawa@bi-lab.org; 4 Institute of Innovative Research, Tokyo Institute of Technology, Tokyo 152-8550, Japan; 5 Office for Academic and Industrial Innovation, Kobe University, Kobe 657-8501, Japan; 6 Brain Impact, Kyoto 606-8501, Japan

**Keywords:** coronavirus disease (COVID-19), social distancing, physical proximity, work characteristics, explanatory factor analysis, O*NET

## Abstract

The coronavirus disease (COVID-19) continues to spread globally. While social distancing has attracted attention as a measure to prevent the spread of infection, some occupations find it difficult to implement. Therefore, this study aims to investigate the relationship between work characteristics and social distancing using data available on O*NET, an occupational information site. A total of eight factors were extracted by performing an exploratory factor analysis: work conditions, supervisory work, information processing, response to aggression, specialization, autonomy, interaction outside the organization, and interdependence. A multiple regression analysis showed that interdependence, response to aggression, and interaction outside the organization, which are categorized as ”social characteristics,” and information processing and specialization, which are categorized as “knowledge characteristics,” were associated with physical proximity. Furthermore, we added customer, which represents contact with the customer, and remote working, which represents a small amount of outdoor activity, to our multiple regression model, and confirmed that they increased the explanatory power of the model. This suggests that those who work under interdependence, face aggression, and engage in outside activities, and/or have frequent contact with customers, little interaction outside the organization, and little information processing will have the most difficulty in maintaining social distancing.

## 1. Introduction

The coronavirus disease (COVID-19) continues to spread steadily. At the time of writing, on 7 April, 2021, there are 132,423,013 infected people worldwide [1]. The recommended practice for preventing infection is social distancing, that is, maintaining a specific physical distance between people [2]. However, it is not easy to practice social distancing in all jobs; hence, the current research was conducted to clarify the relationship between work characteristics and social distancing. We believe that such research can provide valuable implications about, and insights into, the new industrial structure in the post-COVID era. For example, it may be difficult to practice social distancing in a workplace where there is a lot of interaction with people. On the other hand, if the job is to process information using a computer, it will be easy to maintain social distancing. Clarifying what kind of work is easy or difficult to maintain social distancing can provide suggestions for considering the new way of work we should choose after COVID-19. Our attempt is based on growing research to develop the indices related to social distancing and working remotely [3,4,5,6], but this does not mean that the research conducted is problem-free. There is a lack of clarity and validity on how to select the appropriate factors influencing social distancing.

Therefore, the purpose of this study is to find the work characteristics that influence social distancing (physical proximity as a proxy variable) using data available on O*NET, an occupational information site.

## 2. Literature Review and Hypotheses

Research to clarify the relationship between work characteristics and social distancing has been gathering pace [3,4,5,6]. Among the studies on this topic, the Social Distancing Index developed by Koren and Pető [6] and Remote Working Index developed by Dingel and Neiman [4] have 102 and 761 citations, respectively (as of 7 April, 2021, computed by Google Scholar). The Social Distancing Index classifies 14 questionnaire items recorded on O*NET under three factors. The first factor, teamwork, is based on the understanding that some occupations require face-to-face communication several times a week with other workers (e.g., maintenance, personal care-related occupations, and health care professionals). The second factor, customer, is based on the understanding that some occupations require frequent face-to-face contact with customers (e.g., retail salespersons, social workers, and waiters and waitresses). The third factor, presence, is based on the understanding that some occupations require physical proximity to one another even if there is no direct communication, for example, to operate machinery or access key resources (e.g., drivers and machine operators in mining and water transport where crammed working environments are common). These factors aimed to capture the jobs that can be performed less efficiently from home, as validated by the association with the American Time Use Survey, which directly asks about the possibility of working from home [6].

The Remote Working Index also creates variables based on the assumption that the 17 items related to outdoor work, etc., recorded on O*NET, influence the executability of remote work. If any of the conditions in the survey responses are true for an occupation, it is coded as an occupation that cannot be performed at home. The feasibility of working at home for all occupations is classified, and it has been found that 37% of jobs in the United States can be performed entirely at home. In addition, it has also been found that jobs that can be done at home typically pay more, and lower income economies have a lower share of jobs that can be done at home [4].

However, as both indices were constructed not by the psychometrics method but by the authors’ criteria, there may be a shortage of items included in a variable or an overload of items in a variable that should be divided into different ones. For example, regarding the concern on the shortage, previous studies on the conductibility of remote working were based on the assumption that many information-handling or technologically specialized jobs are less required to be done at a particular place than object-handling or multi-tasking jobs, e.g., [5,7]. However, these knowledge-related characteristics were not taken into account in the development of the above-mentioned indices [4,6]. This unbalance may perhaps be because they are not based on a particular work characteristics theory like the one by Morgeson and Humphrey [8]. Another concern, an overload of items in a variable, is observed in teamwork, by Koren and Pető [6], which includes items related to leadership. Perhaps there is a difference in the feasibility of social distancing between the vertical and horizontal teams. Further, there is a problem in the correlation between work context–physical proximity, the items seemingly closest to the feasibility of social distancing and remote working among those recorded on O*NET, and each item that has not been verified.

Therefore, in this study, to eliminate arbitrariness as much as possible, some factors common to various occupations were extracted by performing an exploratory factor analysis. We showed factors that determine physical proximity by performing a multiple regression analysis using the constructed independent variables. At the same time, we analyzed teamwork, customer, and presence from Koren and Pető [6], and remote working from Dingel and Neiman [4] as independent variables, to verify whether these are useful as determinants of social distancing.

According to Morgeson and Humphrey [8], there are four types of work design characteristics: social, knowledge, task, and contextual characteristics. Of these, social characteristics involve relationships with people (interdependence, social support, etc.), which are expected to require physical proximity because “physical proximity has a tremendous impact on the ability to work together” (Kiesler and Cummings, p. 57) [9]. Thus, the following hypothesis was derived:

**Hypothesis** **1** **(H1).** *Interdependence and social support (or another relationship-related interaction which needs proximity to others such as responding to aggression) of social characteristics are positively related to physical proximity*.

Conversely, knowledge characteristics (information processing, specialization, etc.), and some social characteristics (interaction outside the organization, etc.) are in many cases considered to be aspects of jobs that do not require physical contact with people, e.g., [5,7]. For example, the job of information processing will reduce the need for physical proximity because it does not require interaction with people. Thus, the following hypothesis was derived:

**Hypothesis** **2** **(H2).** *Information processing and specialization of knowledge characteristics are negatively related to physical proximity. Interaction outside the organization (H2c) of social characteristics is negatively related to physical proximity*.

We did not hypothesize for the remaining task (autonomy, task significance, etc.), and contextual characteristics (work conditions, equipment use, etc.) because they appear neutral to physical proximity. For instance, it is indicated that manufacturers who work autonomously tend to achieve high performance only if their interdependency level is low [10]. In contrast, salespersons who work autonomously may have to work face-to-face with their customers to achieve high performance. Therefore, it is considered that there is a big difference in physical proximity even in the same-level autonomous conditions by occupations. Likewise, people may be able to perform jobs such as that of food management in a commercial large freezer alone, but it may be necessary to work with multiple people in jobs such as snow removal in cold regions. Therefore, it is considered that there is a big difference in physical proximity even when working in a cold place.

## 3. Methods

We conducted an explanatory factor analysis to extract the factors of work characteristics. Then, we conducted a multiple regression analysis to identify the work characteristics that determine physical proximity. For that purpose, we discuss the source and nature of data in this section.

### 3.1. Samples and Data Collection

We used the responses to two surveys classified by 968 occupations included in release 24.3 of the database administered by O*NET (https://www.onetonline.org/, accessed on 8 August 2020, leased in May 2020. The accessed data were Work Context: https://www.onetonline.org/find/descriptor/browse/Work_Context/, accessed on 8 August 2020 and Work Activities: https://www.onetonline.org/find/descriptor/browse/Work_Activities/, accessed on 8 August 2020), a program sponsored by the US Department of Labor to investigate the association between work characteristics and physical proximity. The first survey is called the Work Context Questionnaire and includes questions about the “physical and social factors that influence the nature of work” such as interpersonal relationships, physical work conditions, and structural job characteristics. The second survey is called the Generalized Work Activities Questionnaire and includes questions about the “general types of job behaviors occurring on multiple jobs” such as the input of information, interaction with others, mental processes, and work output. We used all 98 items recorded under these questionnaires. Importance and level are recorded under Work Activities, but in this study, importance was used according to previous research for optimum clarity. All items take a number from 0 to 100, indicating frequency and importance.

### 3.2. Measures

Physical proximity used as a dependent variable in this study was selected from “I don’t work near other people (beyond 100 ft),” “I work with others but not closely (e.g., private office),” “Slightly close (e.g., shared office),” “Moderately close (at arm’s length),” and “Very close (nearly touching)” for the question “To what extent does this job require the worker to perform job tasks in close physical proximity to other people?” It was assigned a value from 0 to 100 when totaling. Details of other questions and options are available on O*NET; therefore, they are omitted in this paper. However, to give an example, “Work Context—Very Hot or Cold Temperatures,” is “How often does this job require working in very hot (>90 °F) or very cold (<32 °F) temperatures?” The options are “Never,” “Once a year or more but not every month,” “Once a month or more but not every week,” “Once a week or more but not every day,” and “Every day,” with 0, 25, 50, 75, and 100 points assigned to each, to calculate the average value for each occupation. 

### 3.3. Analytical Method

We performed an exploratory factor analysis to extract items to construct variables for the regression analysis. The criterion for factor extraction was an eigenvalue of 1 or more, and the factor load was calculated after performing varimax rotation using the main factor method. After that, items with a factor load of less than 0.4 and of 0.4 or higher on a plurality of factors were excluded, and factor analysis was performed again using the same criteria. This process was repeated until there were no items whose factor loads were less than 0.4 and 0.4 or higher on plural factors. Here, we followed the idea of Stevens [11], who suggests using a cut-off of 0.4, irrespective of sample size, for interpretative purposes. After establishing the factor structure, which is composed of work conditions, supervisory work, information processing, response to aggression, specialization, autonomy, interaction outside the organization, and interdependence, we performed the regression analysis using the variables comprising each factor as the independent variable, and physical proximity as the dependent variable to analyze the factors influencing social distancing.

## 4. Analysis and Results

### 4.1. Exploratory Factor Analysis

As a result of repeating the factor analysis six times using the above method, as shown in Table 1, eight factors comprising 46 items were extracted. Note that the sentences listed are not the questions themselves, but the topic of the questions. Based on the contents of the included items, the factors were named as work conditions, supervisory work, information processing, response to aggression, specialization, autonomy, interaction outside the organization, and interdependence. For these factor names, the terminology of Morgeson and Humphrey [8] was used except for the response to aggression and supervisory work, which were named by the authors as there was a lack of corresponding factor names. Among the factors, interdependence and response to aggression were categorized as “social characteristics,” as per the classification by Morgeson and Humphrey [8]. Likewise, information processing and specialization were categorized as “knowledge characteristics” following the same classification. As hypothesized above, social characteristics variables were positively associated, while knowledge characteristics variables were negatively associated with physical proximity. Interaction outside the organization was categorized as “social characteristics” [8], but considering its nature, it was negatively associated with physical proximity. Autonomy and supervisory work may be categorized as “task characteristics,” and work conditions may be categorized as “contextual characteristics” [8], for which we did not hypothesize, as discussed above. We included supervisory work into “task characteristics” because it is closest to the subcategory “task significance,” which “reflects the degree to which a job influences the lives or work of others, whether inside or outside the organization” (p. 1323) [8].

Physical proximity was not included in any of these variables because it loaded on multiple factors in the first factor analysis. As indicated by the symbol (R) at the end of the sentence, four work conditions overlapped with remote working from Dingel and Neiman [4], and as shown by (P), one of these overlapped with presence from Koren and Pető [6]. Similarly, three of supervisory work and one of interdependence overlapped with teamwork from Koren and Pető [6] as shown by (T). One of response to aggression overlapped with remote working from Dingel and Neiman [4] as shown in (R). There were no duplicate items with customer from Koren and Pető [6]. Overall, nine of the 46 items, or 19.6%, overlapped with those from either or both Dingel and Neiman [4], and Koren and Pető [6].

Table 2 presents the descriptive statistics. In addition to the above eight variables, physical proximity, teamwork, customer, and presence from Koren and Pető [6], and remote working from Dingel and Neiman [4] are listed with their values and correlations as per the authors’ calculation. In general, 0.7 or higher is considered an acceptable reliability coefficient [12], but several researchers have accepted 0.6 or more [13,14]. According to the latter criterion, all eight variables were acceptable, but according to the former, specialization and interdependence were unacceptable. Therefore, we prepared a three-item version without work context: time pressure for specialization, and a three-item version with work context: coordinate or lead others for interdependence, which had been dropped in the first factor analysis for being the same as the other two items but loaded on other factors. After confirming that the reliability coefficients of these variables were 0.7 or higher, they were used for the following analysis alternatively (the result is omitted because it did not make a large difference).

Looking at the magnitude of the correlation coefficient, all except supervisory work and specialization showed a statistically significant correlation with physical proximity at the 5% level. Interdependence (r = 0.31, *p* < 0.01), response to aggression (r = 0.46, *p* < 0.01), and work conditions (r = 0.07, *p* < 0.05) were positively correlated, while information processing (r = −0.09, *p* < 0.01), interaction outside the organization (r = −0.06, *p* < 0.05), and autonomy (r = −0.13, *p* < 0.01) were negatively correlated with physical proximity. However, specialization (r = 0.00, *p* > 0.05), where a negative correlation was expected, and supervisory work (r = 0.06, *p* > 0.05) were not correlated with physical proximity at the 5% level. Among them, values exceeding 0.1, which is the standard value for “small” by Cohen [15], were observed in three variables: interdependence (r = 0.31, *p* < 0.01), response to aggression (r = 0.46, *p* < 0.01), and autonomy (r = −0.13, *p* < 0.05). Of these, only interdependence and response to aggression exceeded the standard value of 0.3 for “medium” by Cohen [15].

Moreover, as shown in Table 2, among the three from Koren and Pető [6], customer (r = 0.42, *p* < 0.01) and presence (r = 0.20, *p* < 0.01) exceeded 0.1, with only customer exceeding 0.3. Dingel and Neiman’s remote working [4] (r = −0.36, *p* < 0.01) also exceeded 0.3. However, the coefficient of Koren and Pető’s teamwork [6] (r = 0.10, *p* < 0.01) was below 0.1. This is probably because, as described at the beginning, items that represent interdependence and those that represent supervisory work are mixed in the variable.

### 4.2. Multiple Regression Analysis

The first column of Table 3, Model 1, shows the results of the multiple regression analysis using eight variables by the stepwise method. Interdependence (β = 0.29, *p* < 0.01) and response to aggression (β = 0.46, *p* < 0.01) were positively related to physical proximity, supporting H1, and information processing (β = −0.10, *p* < 0.01), specialization (β = −0.10, *p* < 0.01), and interaction outside the organization (β = −0.25, *p* < 0.01) were negatively related to physical proximity, supporting H2. Among them, specialization was not significantly correlated with physical proximity by simple correlation, but negatively related to physical proximity at a significant level by multiple regression after controlling the effects of other variables. On the other hand, work conditions and autonomy were positively and negatively correlated with physical proximity in simple correlation, respectively, but did not significantly relate to physical proximity in multiple regression after controlling the effects of other variables. Supervisory work was not significantly related to physical proximity in either simple correlation or multiple regression. These three variables are therefore omitted from Table 3.

The second column, Model 2, shows the results of the multiple regression analysis using three variables from Koren and Pető [6] by the stepwise method. Customer (β = 0.60, *p* < 0.01) and presence (β = 0.33, *p* < 0.01) were positively related to physical proximity, the same as in simple correlation, but teamwork (β = −0.18, *p* < 0.01) was negatively related to physical proximity. It is theoretically difficult to think that teamwork is negatively related to physical proximity, so it can be interpreted as a problem of multicollinearity. This indicates that some caution is required when using the three items from Koren and Pető [6]. Therefore, the 14 items that composed the three variables were simply averaged and subtracted from 100 to create one variable Social Distancing Index (α = 0.73, mean value = 50.75, standard deviation = 9.09) following the method of Crowley and Doran [4], and the relationship with physical proximity was examined. Although the problem of multicollinearity was solved, the adjusted R-squared value dropped significantly (β = 0.44, *p* < 0.01, R^2^ = 0.19).

Therefore, in the third column, Model 3, the results of a regression analysis performed on a variable group in which customer and remote working, whose correlation coefficient exceeds 0.3—the “medium” level [15]—are added to Model 1, are shown. In addition to customer and remote working, four of the five variables shown in Model 1, except specialization, were significant. However, the magnitude of the partial regression coefficient was different between the first and third columns, and it seems that there was a problem of multicollinearity. This is understandable considering that many of the items that make up customer and remote working were excluded during the exploratory factor analysis due to the load on multiple factors. Despite this problem, the fact that the variables in the first column became significant even after controlling for the impact of customer and remote working indicates the robustness of Model 1. For reference, the adjusted R-squared values for all three models in Table 3—0.32, 0.29, and 0.49—exceed 0.26, the “large” level [15]. Among them, the value of the mixed model in Model 3 is the highest. Therefore, although it is worrisome that the distortion of the partial correlation coefficient impairs the accuracy of the influence of individual variables, the mixed model in Model 3 is considered to be useful for predicting the feasibility of social distancing more accurately by considering differences in customer contact and outdoor activities.

The occupations listed in Table A1 indicate that the values of physical proximity are within the bottom 100, while those listed in Table A2 indicate that they are within the top 100 among 968 occupations. That is, the occupations included in Table A1 are the top 100 where it is easy to perform social distancing, and the occupations included in Table A2 are the top 100 where it is difficult to perform social distancing. Table A1 comprises what are called experts, including managers, specialists, engineers, scientists, designers, etc., while in Table A2, there are kindergarten and special education teachers, therapists, technicians, medical/dental/surgical assistants, first-line supervisors, barbers/hairdressers, flight/transportation attendants, etc. In other words, Table A1 seeks to include highly educated, high income professionals who handle information in the office, while Table A2 seeks to include interpersonal or customer-contact workers, sometimes with low income and low education levels.

## 5. Discussion

This study aimed to find variables that correlate with physical proximity via an exploratory method. The factors extracted by performing an exploratory factor analysis were work conditions, supervisory work, information processing, response to aggression, specialization, autonomy, interaction outside the organization, and interdependence. The results of the multiple regression analysis support the hypotheses that social characteristics (interdependence, response to aggression, and interaction outside the organization) and knowledge characteristics (information processing and specialization) are associated with physical proximity. Further, we added customer [6], which represents contact with the customer, and remote working [4], which represents a small amount of outdoor activity, to our multiple regression model, finding that the mixed model can be used with higher accuracy when predicting the feasibility of social distancing in the workplace.

In the model presented herein, interdependence, response to aggression, and customer showed a positive correlation, while information processing, interaction outside the organization, and remote working showed a negative correlation with physical proximity. Among them, interdependence was associated with physical proximity because face-to-face communication is required in many situations even today despite the advancement of information and communication technology. This result is reasonable considering what is happening every day in the business field. For instance, who takes the initiative depends on the tasks that change daily; and therefore, when someone performs many tasks, adjustments are frequently made to reduce the number of tasks. Likewise, response to aggression was correlated with physical proximity because collaboration with a person who has poor emotional control is likely to induce an unexpected situation, so it is considered necessary to have such people nearby and to support them mentally cooperating with others around them. Previous studies have shown that maintaining physical proximity with students, for example, is necessary for teachers to turn their negative consciousness into a positive one [16]. A similar situation may occur between a person engaged in some occupations (therapists, flight attendants, etc.) and a customer. Alternatively, it is possible to interpret the causal relationship in reverse. That is, the higher the physical proximity, the greater the chance of being exposed to aggression. A service industry that deals with customers is a typical example. The high correlation between customer and response to aggression observed in Table 2 also supports the existence of such a relationship. However, unfortunately, it seems unlikely that science and technology will immediately replace human contact. For instance, in the field of robot development, it has been established that the most difficult task is to operate in an unstructured environment that assumes contact with humans [17].

Among the variables we used, supervisory work had the weakest correlation. However, this is not unreasonable once we imagine that supervisory work can be demonstrated over long distances depending on the content. For instance, a study showed that virtual is more effective than face-to-face for some kinds of supervisory work [18]. In the same vein, it has been discussed that effective supervisory work is possible even in a virtual environment by creating an atmosphere in which subordinates can easily express their opinions [19]. Similarly, autonomy and work conditions did not show a significant correlation with physical proximity in the multiple regression analysis. This result is also reasonable considering that autonomy or the harshness of work conditions (e.g., very hot or cold temperatures) is not always based on human proximity. Although it has been argued that granting high autonomy and a good environment makes it easier for employees to perform virtual jobs [19], this is limited to jobs that are technically manageable remotely. In other words, the effect of task or contextual characteristics on physical proximity will not outweigh the effect of social or knowledge characteristics.

This study showed that multiple work characteristics correlate with physical proximity. In other words, it becomes more difficult to execute social distancing when a plurality of work characteristics overlaps. A single variable may not have the decisive power to impede/facilitate social distancing. For example, interdependence and response to aggression have a high correlation with physical proximity by itself, but that does not mean that they always require physical proximity. Indeed, it has been shown that by making good use of teleworking, it is possible to strengthen positive relationships and limit negative relationships more than within traditional workplaces [20]. That is, those who work under interdependence, face aggression, and engage in outside activities, have frequent contact with customers, little interaction outside the organization, and little information processing will have the most difficulty in maintaining social distancing. This result gives us an important implication for considering the work after COVID-19. For instance, even for some jobs, which were previously thought to have difficulty social distancing, there may be room to discuss the possibility by carefully analyzing their work characteristics.

## 6. Implication

Our study has three theoretical implications. First, using the results of the factor analysis, we were able to show a model for predicting social distancing (physical proximity). Our model is composed of independent variables, including social characteristics such as interdependence, response to aggression, and interaction outside the organization, and knowledge characteristics such as information processing and specialization. Second, the study has shown that this model is statistically significant without major changes, even when the variables shown in the previous studies, customer and remote working, were added to the independent variables. This indicates the robustness of our model. Third, our model required some modifications to variables that were previously thought to be related to social distancing. For instance, the current research shows that interdependence hinders social distancing, but supervisory work does not, although previous research did not distinguish between them.

## 7. Limitations

First, this paper exploratively extracted the factors that are variables used in the regression analysis, using the average values by occupation from the attitude survey data recorded on the US occupational information site O*NET. Therefore, if the primary data before being aggregated by occupation can be obtained, and the results of this study can be verified, its significance will be immense. Second, physical proximity, used as the dependent variable in the analysis, is a variable based on the questionnaire survey results and may differ from the actual proximity. Therefore, it is also important to verify the analytical model of this study after measuring the actual proximity using GPS location information, etc. Third, we used the term “social distancing” for the model predicting social proximity. Jobs with physical proximity at present may not always be the ones that are difficult with regard to social distancing. In the future, the relationship between physical proximity and social distance should also be examined in a more detailed analysis.

## 8. Recommendation for Future Research

An analysis using the original data of O*NET and research to clarify the relationship between work characteristics and more objective data will be of great significance in the verification and development of the current research.

## 9. Conclusions

COVID-19 is still raging; hence, social distancing has attracted attention as a measure to prevent the spread of infection. However, some occupations find implementation problematic. Therefore, in previous studies, the scale of factors determining social distancing has been developed. Referring to these achievements, this study extracted eight factors by performing an exploratory factor analysis: work conditions, supervisory work, information processing, response to aggression, specialization, autonomy, interaction outside the organization, and interdependence. Of these, interdependence, response to aggression, and interaction outside the organization, which were categorized as social characteristics, and information processing and specialization, which were categorized as knowledge characteristics, correlated with physical proximity, thus, supporting the hypotheses. Further, we added customer, which represents contact with the customer, and remote working, which represents a small amount of outdoor activity, to our multiple regression model and confirmed that they increased the explanatory power of the model, suggesting that this mixed model can be used with higher accuracy when predicting the feasibility of social distancing in the workplace. This study indicates the necessity to focus on various work characteristics to maintain social distancing.

## Figures and Tables

**Table 1:** Results of the exploratory factor analysis.

Item	Work Conditions	Supervisory Work	Information Processing	Response to Aggression	Specialization	Autonomy	Interaction Outside the Organization	Interdependence
1.1. Work Context: Very Hot or Cold Temperatures	***0.92***	0.00	−0.11	0.03	0.01	−0.10	0.01	−0.13
1.2. Work Context: Extremely Bright or Inadequate Lighting	***0.88***	−0.09	−0.02	0.10	0.05	−0.05	−0.04	0.04
1.3. Work Activities: Operating Vehicles, Mechanized Devices, or Equipment (P) (R)	***0.86***	0.03	−0.04	0.09	0.05	−0.02	−0.02	−0.19
1.4. Work Context: Outdoors, Exposed to Weather (R)	***0.86***	0.04	0.01	0.12	−0.16	0.02	0.33	−0.06
1.5. Work Context: Exposed to Hazardous Equipment	***0.85***	−0.01	−0.11	−0.12	0.20	−0.01	−0.26	−0.05
1.6. Work Context: Exposed to High Places	***0.83***	−0.01	−0.03	−0.10	0.02	−0.02	0.03	0.27
1.7. Work Context: Indoors, Not Environmentally Controlled	***0.83***	0.05	−0.04	−0.09	0.02	0.00	0.01	−0.05
1.8. Work Context: In an Open Vehicle or Equipment	***0.82***	0.02	−0.08	−0.08	−0.02	−0.04	−0.01	−0.03
1.9. Work Context: Spend Time Climbing Ladders, Scaffolds, or Poles	***0.79***	−0.01	−0.08	−0.13	−0.03	−0.03	0.02	0.26
1.10. Work Context: Exposed to Minor Burns, Cuts, Bites, or Stings (R)	***0.78***	−0.04	−0.23	0.00	0.07	−0.02	−0.26	−0.13
1.11. Work Context: Outdoors, Under Cover (R)	***0.77***	0.07	0.04	0.02	−0.16	0.05	0.36	0.02
1.12. Work Context: Exposed to Whole Body Vibration	***0.76***	−0.03	−0.07	−0.02	−0.02	−0.05	−0.11	0.08
1.13. Work Context: Sounds, Noise Levels Are Distracting or Uncomfortable	***0.72***	−0.06	−0.13	0.13	0.27	−0.14	−0.30	0.07
1.14. Work Context: Spend Time Keeping or Regaining Balance	***0.71***	−0.02	−0.22	0.16	−0.11	−0.08	−0.14	0.08
1.15. Work Context: Indoors, Environmentally Controlled	***−0.70***	0.12	0.24	0.04	0.13	0.11	0.13	0.17
1.16. Work Context: Work Schedules	***0.47***	−0.00	−0.12	−0.10	−0.28	0.07	0.16	−0.09
2.1. Work Activities: Guiding, Directing, and Motivating Subordinates (T)	0.05	***0.89***	0.19	0.05	−0.02	0.06	−0.00	0.11
2.2. Work Activities: Coordinating the Work and Activities of Others (T)	0.07	***0.86***	0.19	0.07	−0.01	−0.01	0.07	0.19
2.3. Work Activities: Staffing Organizational Units	−0.09	***0.83***	0.14	0.07	0.04	0.06	0.17	−0.02
2.4. Work Activities: Developing and Building Teams (T)	−0.06	***0.82***	0.26	0.17	−0.06	−0.07	0.06	0.21
2.5. Work Activities: Coaching and Developing Others	−0.07	***0.80***	0.24	0.16	−0.19	0.07	−0.19	0.13
2.6. Work Activities: Monitoring and Controlling Resources	0.04	***0.73***	0.10	−0.08	0.10	0.17	0.24	−0.11
2.7. Work Activities: Scheduling Work and Activities	−0.04	***0.68***	0.31	0.01	−0.09	0.24	0.19	−0.03
2.8. Work Activities: Training and Teaching Others	−0.01	***0.62***	0.32	0.09	−0.20	0.06	−0.30	0.09
2.9. Work Activities: Judging the Qualities of Things, Services, or People	0.04	***0.54***	0.34	0.10	0.03	0.17	−0.15	−0.14
3.1. Work Activities: Analyzing Data or Information	−0.26	0.26	***0.79***	−0.18	0.07	0.11	0.10	0.05
3.2. Work Activities: Processing Information	−0.25	0.21	***0.79***	−0.10	0.26	0.01	0.10	0.00
3.3. Work Activities: Interpreting the Meaning of Information for Others	−0.28	0.35	***0.76***	0.01	−0.14	0.15	0.02	0.13
3.4. Work Activities: Updating and Using Relevant Knowledge	−0.26	0.30	***0.74***	−0.03	−0.01	0.24	0.07	0.08
3.5. Work Activities: Getting Information	−0.20	0.22	***0.72***	0.06	0.14	0.14	0.08	0.11
3.6. Work Activities: Documenting/Recording Information	−0.21	0.21	***0.70***	0.19	0.15	0.12	0.12	−0.00
3.7. Work Activities: Identifying Objects, Actions, and Events	0.14	0.24	***0.68***	0.10	0.11	0.09	−0.14	0.02
3.8. Work Activities: Evaluating Information to Determine Compliance with Standards	0.10	0.31	***0.55***	0.16	0.33	−0.09	0.05	0.07
4.1. Work Context: Deal With Unpleasant or Angry People	−0.07	0.08	−0.06	***0.89***	0.20	−0.04	0.05	0.06
4.2. Work Context: Deal With Physically Aggressive People (R)	0.05	0.09	0.06	***0.82***	−0.05	−0.03	−0.04	−0.01
4.3. Work Context: Frequency of Conflict Situations	−0.04	0.28	0.11	***0.74***	0.20	0.13	0.13	0.21
5.1. Work Context: Importance of Repeating Same Tasks	−0.02	−0.15	0.06	0.20	***0.69***	−0.12	0.05	0.02
5.2. Work Context: Importance of Being Exact or Accurate	−0.08	−0.12	0.27	−0.04	***0.69***	0.06	−0.06	0.03
5.3. Work Context: Degree of Automation	−0.05	−0.05	0.03	−0.00	***0.52***	−0.27	0.08	−0.02
5.4. Work Context: Time Pressure	0.08	0.07	0.08	0.06	***0.46***	0.09	−0.04	0.09
6.1. Work Context: Freedom to Make Decisions	−0.07	0.17	0.27	0.02	−0.14	***0.82***	0.06	0.01
6.2. Work Context: Structured versus Unstructured Work	−0.21	0.21	0.24	−0.05	−0.07	***0.77***	0.13	0.04
7.1. Work Context: Telephone	−0.19	0.20	0.31	0.19	0.18	0.39	***0.60***	0.24
7.2. Work Activities: Communicating with Persons Outside Organization	−0.25	0.35	0.40	0.17	−0.09	0.25	***0.52***	0.03
8.1. Work Context: Work With Work Group or Team (T)	−0.01	0.36	0.17	0.27	0.21	−0.10	0.04	***0.50***
8.2. Work Context: Face-to-Face Discussions	−0.00	0.25	0.22	0.16	0.15	0.22	0.07	***0.47***

Note(s): If the factor load is 0.4 or higher, italic and bold type. (T) and (P) indicate that they overlap with teamwork and presence from Koren and Pető [6]. (R) indicates that they overlap with remote working from Dingel and Neiman [4].

**Table 2:** Descriptive statistics.

	Mean	SD	α	1	2	3	4	5	6	7	8	9	10	11	12
1. Physical proximity	60.30	16.88													
2. Interdependence	84.35	8.90	0.66	0.31 **											
3. Response to aggression	36.75	12.88	0.87	0.46 **	0.37 **										
4. Information processing	66.13	11.94	0.92	−0.09 **	0.39 **	0.15 **									
5. Specialization	58.96	9.70	0.69	0.00	0.19 **	0.19 **	0.20 **								
6. Interaction outside the organization	67.57	18.63	0.81	−0.06 *	0.39 **	0.31 **	0.59 **	0.08 **							
7. Autonomy	76.92	11.44	0.90	−0.13 **	0.16 **	0.04	0.42 **	−0.14 **	0.53 **						
8. Supervisory work	48.44	11.81	0.94	0.06	0.44 **	0.24 **	0.55 **	−0.08 **	0.45 **	0.35 **					
9. Work conditions	18.54	17.54	0.93	0.07 *	−0.05	−0.03	−0.31 **	−0.04	−0.31 **	−0.21 **	−0.06				
10. ^a^ Teamwork	55.41	12.08	0.89	0.10 **	0.62 **	0.29 **	0.59 **	−0.02	0.49 **	0.29 **	0.93 **	−0.09 **			
11. ^a^ Customer	53.84	14.10	0.78	0.415 **	0.37 **	0.58 **	0.41 **	−0.07 *	0.63 **	0.36 **	0.53 **	−0.31 **	0.53 **		
12. ^a^ Presence	38.20	19.35	0.91	0.20 **	−0.06	−0.06 *	−0.26 **	0.08 *	−0.40 **	−0.27 **	−0.04	0.81 **	−0.11 **	−0.26 **	
13. ^b^ Remote Working	57.77	13.83	0.91	−0.36 **	−0.06	−0.15 **	0.18 **	−0.04	0.26 **	0.18 **	−0.07 *	−0.84 **	0.01	−0.03	0.92 **

Note(s): *n* = 968; * significance at the 5% level; ** significance at the 1% level; ^a^ is from Koren and Pető [6]; ^b^ is from Dingel and Neiman [4].

**Table 3:** Results of the multiple regression analysis with physical proximity as the dependent variable.

		β	
Variable	Model 1	Model 2	Model 3
Eight variables obtained from the factor analysis			
Interdependence	0.29 **	-	0.25 **
Response to aggression	0.46 **	-	0.16 **
Information processing	−0.10 **	-	−0.16 **
Specialization	−0.10 **	-	
Interaction outside the organization	−0.25 **	-	−0.42 **
Four variables obtained from previous studies			
Teamwork	-	−0.18 **	-
Customer	-	0.60 **	0.57 **
Presence	-	0.33 **	-
Remote Working		-	−0.20 **
R^2^	0.32	0.30	0.49
Adjusted R^2^	0.32	0.29	0.49
F	91.29 **	134.16 **	155.44 **

Note(s): *n* = 968; * significance at the 5% level; ** significance at the 1% level; “-” indicates that it is not used in the regression model; blank cells indicate not selected in stepwise regression.

## Data Availability

Publicly available datasets were analyzed in this study. This data can be found here: (https://www.onetonline.org/, accessed on 8 August 2020, leased in May 2020. The accessed data were Work Context: https://www.onetonline.org/find/descriptor/browse/Work_Context/, accessed on 8 August 2020 and Work Activities https://www.onetonline.org/find/descriptor/browse/Work_Activities/, accessed on 8 August 2020).

## Abbreviation

COVID-19: coronavirus disease.

## Appendix A

**Table A1:** Top 100 occupations with low physical proximity.

Public Relations and Fundraising Managers; Computer and Information Systems Managers; Treasurers and Controllers; Geothermal Production Managers; Purchasing Managers; Compensation and Benefits Managers; Human Resources Managers; Education Administrators, Postsecondary; Natural Sciences Managers; Investment Fund Managers; Purchasing Agents, Except Wholesale, Retail, and Farm Products; Insurance Appraisers, Auto Damage; Equal Opportunity Representatives and Officers; Human Resources Specialists; Management Analysts; Fundraisers; Compensation, Benefits, and Job Analysis Specialists; Market Research Analysts and Marketing Specialists; Accountants; Appraisers, Real Estate; Personal Financial Advisors; Loan Counselors; Computer and Information Research Scientists; Computer Programmers; Computer Network Architects; Web Administrators; Business Intelligence Analysts; Actuaries; Mathematicians; Operations Research Analysts; Statisticians; Biostatisticians; Geodetic Surveyors; Water/Wastewater Engineers; Petroleum Engineers; Wind Energy Engineers; Zoologists and Wildlife Biologists; Bioinformatics Scientists; Epidemiologists; Astronomers; Environmental Scientists and Specialists, Including Health; Climate Change Analysts; Industrial Ecologists; Geoscientists, Except Hydrologists and Geographers; Hydrologists; Remote Sensing Scientists and Technologists; Economists; Environmental Economists; Survey Researchers; Industrial-Organizational Psychologists; Sociologists; Political Scientists; Lawyers; Judicial Law Clerks; Paralegals and Legal Assistants; Engineering Teachers, Postsecondary; Agricultural Sciences Teachers, Postsecondary; Forestry and Conservation Science Teachers, Postsecondary; Chemistry Teachers, Postsecondary; Psychology Teachers, Postsecondary; Social Work Teachers, Postsecondary; History Teachers, Postsecondary; Craft Artists; Fine Artists, Including Painters, Sculptors, and Illustrators; Graphic Designers; Talent Directors; Music Composers and Arrangers; Poets, Lyricists and Creative Writers; Pathologists; Parking Enforcement Workers; Cooks, Private Household; Pesticide Handlers, Sprayers, and Applicators, Vegetation; Motion Picture Projectionists; Sales Engineers; Payroll and Timekeeping Clerks; Meter Readers, Utilities; Executive Secretaries and Executive Administrative Assistants; First-Line Supervisors of Logging Workers; Animal Breeders; Agricultural Equipment Operators; Nursery Workers; Farmworkers and Laborers, Crop; Hunters and Trappers; Fallers; Logging Equipment Operators; Electronic Home Entertainment Equipment Installers and Repairers; Camera and Photographic Equipment Repairers; Food and Tobacco Roasting, Baking, and Drying Machine Operators and Tenders; Pressers, Textile, Garment, and Related Materials; Sewers, Hand; Crushing, Grinding, and Polishing Machine Setters, Operators, and Tenders; Mixing and Blending Machine Setters, Operators, and Tenders; Cutters and Trimmers, Hand; Cleaning, Washing, and Metal Pickling Equipment Operators and Tenders; Bridge and Lock Tenders; Conveyor Operators and Tenders; Dredge Operators; Excavating and Loading Machine and Dragline Operators; Industrial Truck and Tractor Operators; Wellhead Pumpers; Refuse and Recyclable Material Collectors.

Note(s): The occupations listed in Table A1 indicate that the values of physical proximity are within the bottom 100 of 968.

**Table A2:** Top 100 occupations with high physical proximity.

Kindergarten Teachers, Except Special Education; Special Education Teachers, Preschool; Adapted Physical Education Specialists; Teacher Assistants; Actors; Dancers; Choreographers; Singers; Chiropractors; Dentists, General; Oral and Maxillofacial Surgeons; Orthodontists; Prosthodontists; Optometrists; Anesthesiologists; Family and General Practitioners; Internists, General; Obstetricians and Gynecologists; Pediatricians, General; Surgeons; Allergists and Immunologists; Dermatologists; Hospitalists; Ophthalmologists; Physical Medicine and Rehabilitation Physicians; Sports Medicine Physicians; Urologists; Physician Assistants; Anesthesiologist Assistants; Podiatrists; Occupational Therapists; Low Vision Therapists, Orientation and Mobility Specialists, and Vision Rehabilitation Therapists; Physical Therapists; Radiation Therapists; Recreational Therapists; Music Therapists; Respiratory Therapists; Exercise Physiologists; Veterinarians; Registered Nurses; Acute Care Nurses; Critical Care Nurses; Nurse Anesthetists; Nurse Midwives; Nurse Practitioners; Acupuncturists; Naturopathic Physicians; Orthoptists; Dental Hygienists; Cardiovascular Technologists and Technicians; Diagnostic Medical Sonographers; Nuclear Medicine Technologists; Radiologic Technologists; Emergency Medical Technicians and Paramedics; Psychiatric Technicians; Respiratory Therapy Technicians; Surgical Technologists; Veterinary Technologists and Technicians; Ophthalmic Medical Technicians; Licensed Practical and Licensed Vocational Nurses; Orthotists and Prosthetists; Neurodiagnostic Technologists; Ophthalmic Medical Technologists; Radiologic Technicians; Surgical Assistants; Athletic Trainers; Midwives; Psychiatric Aides; Nursing Assistants; Orderlies; Occupational Therapy Aides; Physical Therapist Assistants; Physical Therapist Aides; Massage Therapists; Dental Assistants; Medical Assistants; Veterinary Assistants and Laboratory Animal Caretakers; Municipal Firefighters; Transportation Security Screeners; First-Line Supervisors of Food Preparation and Serving Workers; First-Line Supervisors of Personal Service Workers; Gaming Dealers; Gaming and Sports Book Writers and Runners; Barbers; Hairdressers, Hairstylists, and Cosmetologists; Makeup Artists, Theatrical and Performance; Manicurists and Pedicurists; Shampooers; Skincare Specialists; Personal Care Aides; Cement Masons and Concrete Finishers; Pipelayers; Structural Iron and Steel Workers; Rail-Track Laying and Maintenance Equipment Operators; Derrick Operators, Oil and Gas; Roustabouts, Oil and Gas; Electrical and Electronics Repairers, Powerhouse, Substation, and Relay; Meat, Poultry, and Fish Cutters and Trimmers; Flight Attendants; Ambulance Drivers and Attendants, Except Emergency Medical Technicians; Transportation Attendants, Except Flight Attendants.

Note(s): The occupations listed in Table A2 indicate that the values of physical proximity are within the top 100 of 968.
